# Time-resolved characteristics of laser induced breakdown spectroscopy on non-flat samples by single beam splitting

**DOI:** 10.1039/d0ra06582j

**Published:** 2020-10-28

**Authors:** Bingying Lei, Boping Xu, Jing Wang, Jing Li, Yishan Wang, Jie Tang, Wei Zhao, Yixiang Duan

**Affiliations:** State Key Laboratory of Transient Optics and Photonics, Xi'an Institute of Optics and Precision Mechanics of CAS Xi'an 710119 China tangjie@opt.ac.cn; School of Future Technology, University of Chinese Academy of Sciences Beijing 100049 China; Key Laboratory of Synthetic and Natural Functional Molecule Chemistry of Ministry of Education, College of Chemistry and Materials Science, Northwest University Xi'an 710127 China

## Abstract

A single-beam-splitting approach was used to enhance the signal intensity of LIBS under the extreme conditions of laser beam grazing of the surface of non-flat samples. Time-resolved spectra show that the laser-ablated plasma presents a stronger spectral intensity and a slower plasma decay in the split beam mode because of the higher laser irradiance. The temporal evolutions of signal enhancement factors indicate that the enhancement effect first rises and then drops with delay time and the maximum enhancement factor of Al plasma comes later than that of Cu plasma under the same laser energy. The mechanisms behind it are discussed. It is also found that the electron density exhibits a faster decay with delay time in the split beam mode, mainly due to the faster plasma expansion. And a slower increase of electron density with laser energy is observed in the split beam mode because of the plasma shielding effect.

## Abstract 2

A single-beam-splitting approach was used to enhance the signal intensity of LIBS under the extreme condition of a laser beam grazing the surface of non-flat samples. We investigated the time-resolved characteristics of laser-induced plasma under single- and split beam modes.

## Graphical abstract

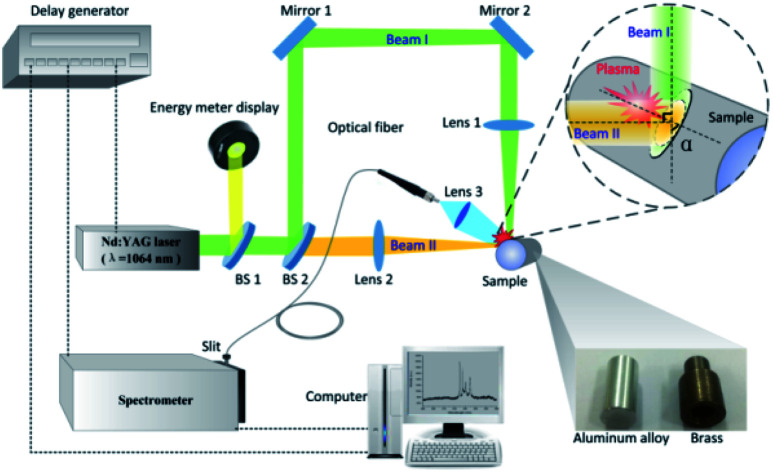

## 1. Introduction

Laser-induced breakdown spectroscopy (LIBS) as a powerful field-deployable tool for elemental analysis has attracted increasing attention over the past decades. LIBS is a spectroscopic analysis technique relying on the optical detection of light emitted from the laser-generated plasma plume to determine the composition of the target.^1–5^ The capability of LIBS to provide rapid, standoff, and multi-element analysis of various samples (solid, liquid, gaseous, or aerosol) without sample preparation has been widely demonstrated.^6–8^ Due to its unique features, LIBS has been recognized as a portable and versatile analytical spectroscopic technique, which has a wide range of potential applications including qualitative and quantitative examination in planetary exploration, industrial applications, explosives detection, and geological analysis.^8–10^

Despite the prominent superiority of LIBS, its low sensitivity and high limit of detection (LoD) remain as the critical limitations of this technique compared to other conventional atomic spectroscopic techniques, such as inductively coupled plasma atomic emission spectrometry (ICP-AES) or inductively coupled plasma mass spectroscopy (ICP-MS).^10^ Various approaches have been undertaken to improve the optical emission spectroscopy (OES) signals for different materials, for example, double or multiple laser pulse excitation, coupling of microwave or fast spark discharge, spatial and/or magnetic confinement, and introduction of vacuum chamber and ambient gas.^12–17^ Another method employed for signal enhancement is the double-pulse LIBS with a single laser system, which emerged as a promising technique due to the advantages of the significant enhancement effect, low cost, and simplicity.^18–21^ This approach has been already proposed earlier by Antony *et al.*, where two laser pulses generated from one laser focus on the same small surface area of specimen of interest.^18^ A double-pulse fs-LIBS with collinear configuration by utilizing a single laser system was reported and the enhancement factor of 4 was observed for Zn atomic lines.^21^ In addition, Piñon and Anglos carried out a comparative study of collinear double-pulse and single-pulse femtosecond LIBS on brass target. A significant increase of the intensity and reproducibility of the optical emission signal was observed when an appropriate interpulse delay was selected.^11^ From the perspective of economy and convenience, ns-laser is more preferable because of its lower cost and smaller size. Yang *et al.* investigated a double-pulse ns-LIBS technique with a new single-beam-splitting scheme. They obtained an enhancement factor up to 5.6 for Al atomic lines. However, when the laser energy was set below 60 mJ, an increase in signal intensity of emission spectra was not obtained.^19^

Although the double-pulse LIBS technique with single laser system has been proven to be effective in laboratory, the complex ambient conditions introduce extreme constraints from the point of view of applicability, which has a negative effect on LIBS measurement.^22^ Among the environmental factors, sample surface is one of our most concerns since it brings a significant impact on the sensitivity and reliability of the LIBS technique, especially for the detection and analysis of trace elements.^6,23^ The irregular targets, *i.e.*, non-flat samples, are common in practical application of LIBS technique. Different from the standard or well-selected sample in laboratory, the surface of non-flat sample is complex and difficult to predict, which may lead to a normal incidence unavailable when the laser pulse ablates the target material. A larger incident angle of laser beam causes a decrease in laser irradiance, which will reduce the ablated mass as well as laser ablation efficiency. As a result, the emission intensity of laser plasma is largely weakened and the sensitivity of LIBS technique is greatly limited. However, little work has been carried out to clarify the influence of sample surface on LIBS measurement. Additionally, few approaches, to our knowledge, have proposed a more sensitive and reliable method for LIBS technique when laser beam grazes the non-flat sample surface. Although the multiple shots accumulation (often hundreds) and multivariate calibration models were used to eliminate the influence of non-flat surface as far as possible, high cost and operational complexity could not be avoided.^24,25^ Moreover, almost no systematic work was performed to more comprehensively reveal the physical mechanisms behind signal enhancement by using the single-beam-splitting scheme under such extreme condition. To fill this gap, in this work, a single-beam-splitting scheme with the advantage of simplicity and low cost is used to improve the signal intensity of LIBS for non-flat samples. Different from the single-beam-splitting method noted above, an obvious enhancement effect on emission intensity is obtained when a 1064 nm Nd:YAG laser with pulse energy below 60 mJ is utilized in our case.^19^ In order to better understand the physical mechanisms behind the signal enhancement, the time-resolved characteristics of laser-induced plasma obtained by single-beam-splitting LIBS and by traditional single beam LIBS are comparatively investigated.

## 2. Experimental

**Fig. 1:**
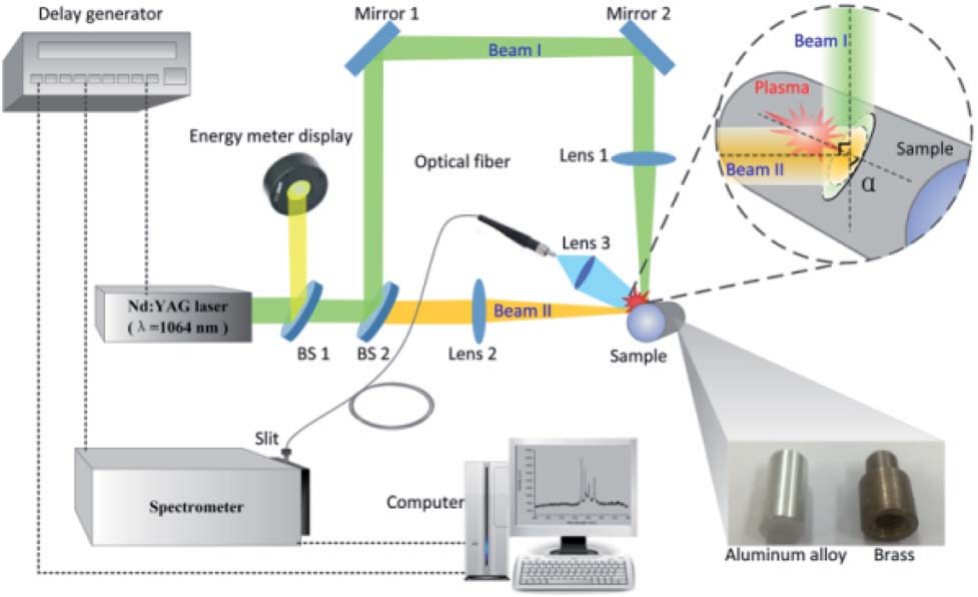
Schematic diagrams of the experimental setup.

Fig. 1 shows the experimental setup for double-pulse LIBS with single-beam-splitting scheme. The ablation laser source was a Q-switched Nd:YAG laser (Quantel, Ultra 100) delivering 8 ns pulse-duration at 1064 nm and was operated at a 20 Hz repetition rate. The output energy was adjusted from 18 to 48 mJ and monitored by the laser energy meter (Nova II) through measuring a small fraction of the incident laser beam separated by a beam-splitter (BS 1, THORLABS BST11). Subsequently, the laser pulse is divided into a reflection and transmission laser beam by a 45 degree beam-splitter (BS 2, THORLABS BST11). After being reflected by mirror 1 and mirror 2, the vertical beam I was tangentially focused on the sample surface by lens 1 with a 15 cm focal length, the energy of which accounts for ∼70% of the total. The angle of incidence (AOI) for beam I is around 85°, which is approximately measured by taking the cross-section image of the processed cylindrical samples and comparing the sample characteristic dimensions. The lens 2 with 15 cm focal length was used to focus the transmission laser beam II (the energy accounts for ∼30% of the total) on the surface of the sample to a spot size ∼ 75 um with nearly normal incident angle. The AOI between beam I and beam II was fixed at 90°. Since the optical path difference (OPD) is around 50 cm, the delay time between the two beams could be estimated and its value equals about 1.5 ns, which is less than 1/5 of the laser pulse width (8 ns). Therefore, it can be considered that these two beams simultaneously ablate the sample surface and then generate the plasma plume in the split beam mode. For the single beam mode, only beam I is applied and its energy is set the same as the total energy in the split beam mode for comparison of the signal enhancement effect. Here, beam I grazes the sample surface, where this incident laser beam deposits all its energy on the side of the cylindrical sample. In addition, the cylindrical samples aluminum alloy and brass (10 mm diameter and 20 mm height) are selected to simulate the non-flat samples. The sample is mounted on a three-dimensional translation platform to ensure that both two beams are incident on the sample target. In our experiment, the vertical beam I is first fixed, and then the lens 2 located on the two-dimensional translation stage is adjusted within a small distance in order to achieve the coincidence of the two laser beam focal points. Each sample was finely polished by using the sandpaper and multiple burn-off pulses were applied before spectra recording. To avoid the over ablation of target, the sample is rotated to ensure a fresh sample surface. The fresh surface is exposed for each measurement including a sequence of spectra at different delay times. The distance between the focusing lens and the sample surface is kept less than the focal length to avoid any breakdown of the air in front of the sample.

The spatially integrated emissions from the laser plasma are collected by a fused-silica optical fiber with core diameter of 100 μm. Prior to the experiment, spectra with higher intensity were obtained when the optical fiber was placed in the plane determined by beam I and beam II, with its intersection angle relative to beam I in the range of 30°–60°. Considering the limited space, the optical fiber is placed at an angle of 45° with respect to beam I for convenience and reliability of experimental operation. And the optical fiber is connected to the entrance slit of a spectrometer system (Andor Tech., Shamrock 303i) to examine the plasma emission spectrum. The spectroscopic collection system is triggered by the Q-switched of Nd:YAG laser. The spectrum range is set as required and the spectrum is corrected by subtracting the dark current of the detector which was obtained separately. The collected emission light is dispersed by a 2400 lines per nm gating and sent to an intensified charged-coupled device (ICCD) camera (Andor Tech., iStar 340T). With the purpose of maximizing the signal-to-background (SBR) and signal-to-noise ratios (SNR) of the emission spectra, the delay time and the gate width are adjusted. It should be noted that the delay time means the ICCD gate delay, rather than the delay between the arms of the dual beam setup. To calibrate the spectral wavelength, a mercury argon lamp is used and the obtained spectral lines are compared with NIST database. For this experiment, 5 measurement results are averaged to obtain a set of time-resolved spectra. All spectra are corrected by subtracting the dark current of the spectrometer.

## 3. Results and discussion

### 3.1 Time-resolved emission spectra

**Fig. 2:**
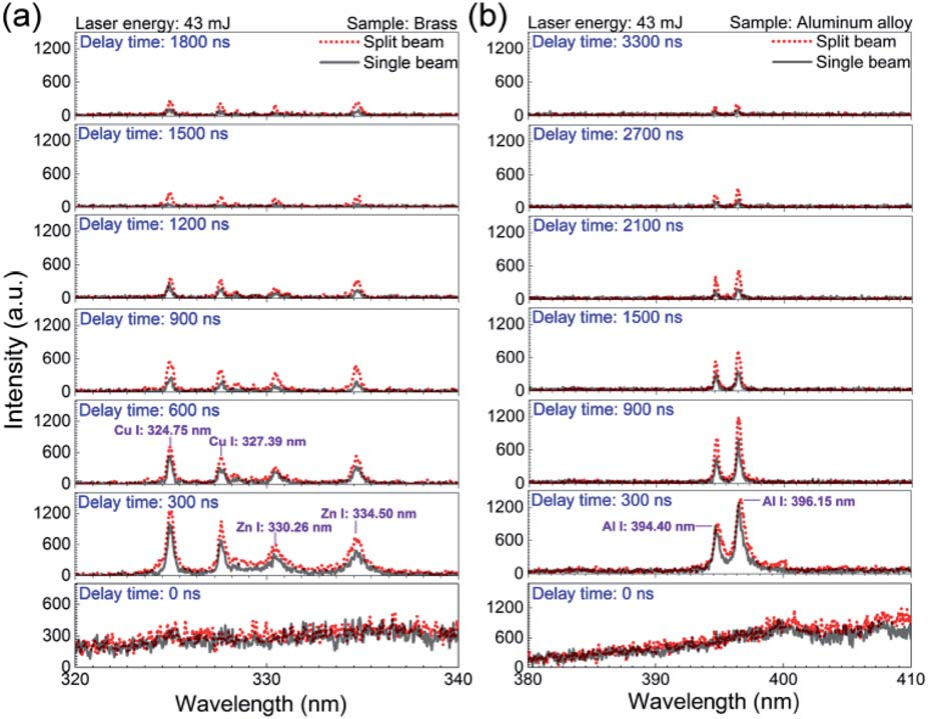
Comparison of the time-resolved emission spectra in the single beam mode and the split beam mode at 43 mJ for (a) the sample brass and (b) aluminium alloy. The gate width is 300 ns.

In an effort to well understand the plasma evolution as well as the enhancement effect with the single-beam-splitting scheme on non-flat sample, the time-resolved spectra were investigated by recording the optical emission spectra with the laser plasma decaying when the laser energy of 43 mJ was used. The gate width and the step were both set at 300 ns unless otherwise stated. The delay time ranges from 0 to 1800 ns for the sample brass and from 0 to 3300 ns for the sample aluminium alloy. Here, the delay time can be considered as the lifetime of the plasma in its decay process. Typical temporal evolutions of the LIBS spectra of brass and aluminium alloy under the split beam and single beam modes are comparatively presented in Fig. 2. In both shooting modes, at the early stage of the plasma expansion (*t* = 0 ns), the plasma emissions are characterized by overwhelming continuum background emission over full range of the radiation, essentially as a result of radiative recombination between electrons and ions (free–bound) and bremsstrahlung emission (free–free).^8,26^ For the case of the sample brass in Fig. 2(a), the background emission spectra are weakened rapidly in the delay time from 0 to 600 ns, and the sharpened atomic lines begin to dominate the spectra. In the following delay time (after 600 ns), the plasma is cooled down in its expansion process. The emission intensity of atomic lines gradually decays and almost becomes invisible at 1800 ns. Comparing the two shooting modes indicates that the emission intensity of atomic lines (Cu I: 324.75 nm, Cu I: 327.39 nm, Zn I: 330.26 nm, and Zn I: 334.50 nm) in the split beam mode is stronger than that in the single beam mode after the delay time of 0 ns, which means the enhancement effect of emission intensity from the sample brass by splitting beam exists almost across all the plasma expansion process. For the case of the sample aluminium alloy in Fig. 2(b), a similar temporal evolution of emission intensities of Al(i) lines (394.40 nm and 396.15 nm) are observed in comparison with the case of the sample brass, except that the spectral lines can even be distinguished at the delay time of 3300 ns, which indicates a longer lifetime for Al plasma.

The optical emission intensity, closely associated with laser ablation efficiency, is largely determined by the laser irradiance, which can be calculated *via I* = *E*/(*S* × *τ*). Here, *E* means the laser beam energy, *τ* is the FWHM of laser pulse and takes the value of 8 ns in our case. *S* is the contact area between the sample surface and the incident laser beam. As depicted in Fig. 1, since the incident angle of vertical beam I (*α*) is measured to be about 85°, the corresponding contact area (*S*) under the single beam and split beam modes can be estimated without consideration of the optical system error.^27^ For instance, with the laser energy set at 33 mJ, the laser irradiance are estimated to be 33.9 GW cm^−2^ and 8.2 GW cm^−2^ for the split beam mode and the single beam mode, respectively. Because 33.9 GW cm^−2^ > 8.2 GW cm^−2^, the higher laser irradiance in the split beam mode leads to a higher laser ablation efficiency, which enlarges the ablated mass and the number of ablated particles. Therefore, the improved emission intensity from laser plasma produced by splitting a single beam can be attributed to more intense heating of laser pulses in the split beam mode.

As for the difference between the lifetime of Al and Cu plasmas under both shooting modes, the possible reason is the difference in the excited energy levels. Assuming local thermodynamics equilibrium (LTE), the emission intensity of spectral line (*I*_mn_), corresponding to a transition from level m to level n, is related to upper state energy (*E*_m_) by the following expression:^28^1
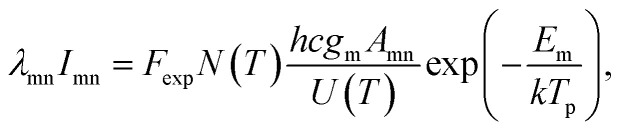
where *λ*_mn_, *h*, *c*, *g*_m_, *A*_mn_ are the wavelength, Plank constant, speed of light in vacuum, statistical weight of upper level of the transition, and transition probability respectively. *k* is the Boltzmann constant, *T*_p_ is the plasma temperature, *U*(*T*) is the partition function, and *N*(*T*) is the total number density of species in plasma. *F*_exp_ represents the detection efficiency for the different elements, considering the collection and detection system, including lenses, optical fiber, spectrograph and ICCD response. The upper state energy for Al(i) 394.4 nm and Cu(i) 324.75 nm are 3.14 eV and 3.82 eV, respectively. From eqn (1), it is observed that the *I*_mn_ is proportional to exp(−*E*_m_/*T*_p_). The decreasing plasma temperature during the plasma cooling process has a more influence on the emission intensity of spectral lines originated from the higher upper state energy, which is probably responsible for the more rapid decay of emission intensity of Cu plasma.^11^

**Fig. 3:**
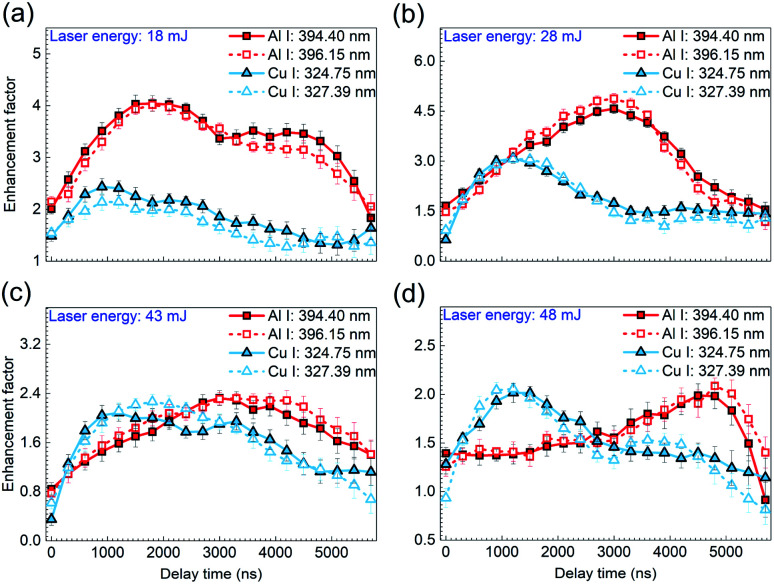
Enhancement factors of atomic lines from the sample aluminium alloy (Al I 394.40 nm and Al I 396.15 nm) and the sample brass (Cu I 324.75 nm and Cu I 327.39 nm) as a function of the delay time at the laser energy of (a) 18 mJ, (b) 28 mJ, (c) 43 mJ, and (d) 48 mJ. The error bar indicates the data deviation within 10 calculations under the same experimental condition.

Since there exists an enhancement of time-resolved emission spectra for different delay times, it is important to go further into the evolutions of enhancement effect during the laser plasma decaying process with laser energy varied. The atomic lines of Al(i) 394.40 nm and Al(i) 396.15 nm from the aluminium alloy as well as Cu(i) 324.75 nm and Cu(i) 327.39 nm from the brass are selected as characteristic spectral lines. The enhancement factor is defined as the ratio of peak intensity under the split beam mode to that in the single beam mode, where the spectral intensity used are extracted from the time-resolved emission spectra at the delay time ranged from 0 to 5800 ns. As shown in Fig. 3, the enhancement factors of spectral intensities from the aluminium alloy and brass are plotted as a function of the delay time with different laser energies of 18, 28, 43, 48 mJ, respectively. Here, five measurement results were averaged to give peak intensity under both the single beam mode and the split beam mode for each calculation of the enhancement factor. And the symbol in each data represents the averaged value of 6 repeated calculations.

For the sample brass, it can be seen from Fig. 3(a) that under the laser energy of 18 mJ, the enhancement factors of both Cu(i) lines first rise till reaching their maximum, and then drop gradually with the plasma decaying. According to the formation mechanisms of ns-laser-induced plasma, the increased laser irradiance in the split beam mode contributes much to the overwhelming background continuum, but little to the atomic lines generated in the early phase of plasma decay, which accounts for the weak enhancement of atomic lines.^5,26^ But with the plasma expanding in the following time, the increase of laser irradiance in the split beam mode are mainly applied to generation of atomic spectra, under which the excitation efficiency is greatly strengthened. Thus, an apparent enhancement effect is obtained. However, with the plasma cooling, a gradual decrease in the enhancement effect is observed in the late phase of plasma decay. With the laser energy increased to 28, 43, 48 mJ, as shown in Fig. 3(b)–(d), a similar variation of enhancement factor with the delay time is presented except that the optimal enhancement performance occurs at the laser energy of 28 mJ.

With respect to the sample aluminium alloy, the temporal evolution of enhancement factor presents a similar tendency under various laser energies, compared to the case of the sample brass. But a distinctive feature is that the delay time at which the maximum enhancement factor is located arrives later under the certain laser energy. It is known that aluminum and copper (especially for different alloys containing these elements are present in this work) have different atomic structure and the emission lines observed in this work are from significantly different energy levels. More specifically, the electron configurations of ground state Al and Cu atoms are 1s^2^2s^2^2p^6^3s^2^3p^1^ and 1s^2^2s^2^2p^6^3s^2^3p^6^3d^10^4s^2^, respectively. The transition from the upper level configuration to the lower level is 3s^2^4 s^12^S_1/2_–3s^2^3p^12^P_3/2_ for Al(i) 396.15 nm and 3d^10^4p^12^P_1/2_–3d^10^4s^12^S_1/2_ for Cu(i) 327.39 nm. Because of these differences, the intrinsic interaction between the laser and samples may allow the Al plasma possessing stronger spectral intensity and longer lifetime under the same laser energy, which leads to the later arrival of the maximum enhancement factor of Al plasma. Additionally, for the sample aluminium alloy, a remarkable characteristic is that with the laser energy increasing, the delay time at which the maximum enhancement factor appears is postponed, *i.e.*, the maximum enhancement factors for Al(i) 394.40 nm and Al(i) 396.15 nm appear around 1200 ns, 3000 ns, 4000 ns, and 5000 ns for the laser energy of 18 mJ, 28 mJ, 43 mJ, 48 mJ, respectively. It is generally accepted that the increasing laser energy ablates more mass from the sample and makes the laser plasma last a longer time. This behavior not only increases the intensity of characteristic spectral line and prolongs its lifetime in the plasma expansion process, but also influences ionization balance and the charge state in the laser plasma, which possibly gives rise to that the optimal enhancement of emission intensity due to the beam splitting arrives later.^29,30^

### 3.2 Plasma properties: excitation temperature (*T*_p_) and electron density (*N*_e_)

The plasma temperature (*T*_p_) and electron density (*N*_e_) are two important physical parameters in characterization of laser plasmas, which can be used in well understanding the mixing behavior between emitting species.^3,30^ Assuming that the plasma is in Local Thermodynamic Equilibrium (LTE), excited species follow the Boltzmann distribution. By transforming eqn (1), the plasma temperature can be determined using the following relation:2
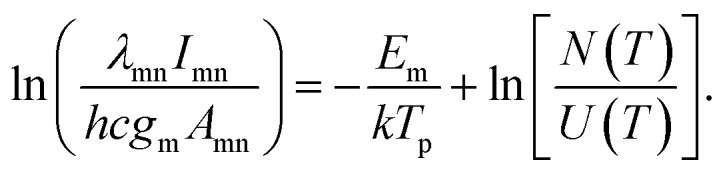


**Fig. 4:**
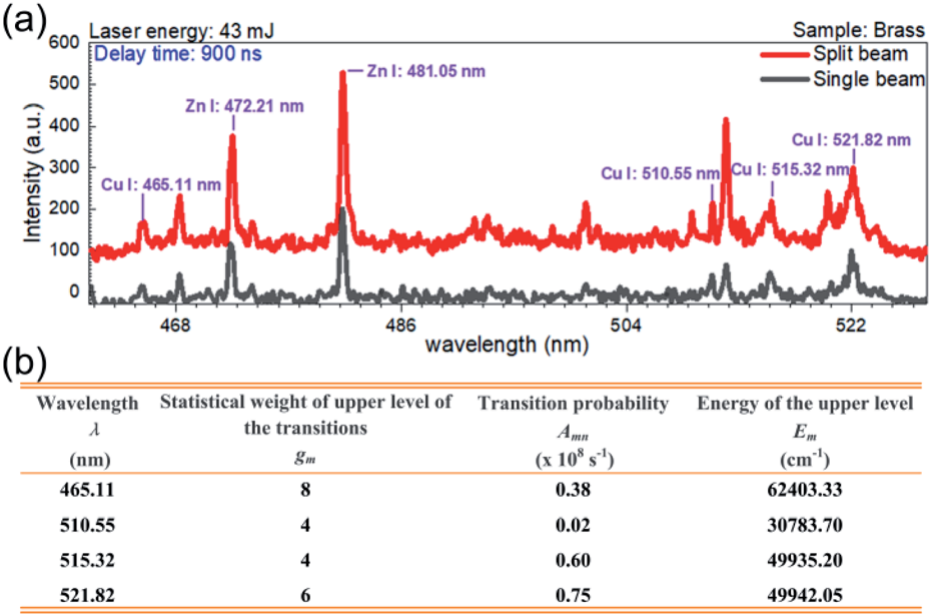
(a) Comparison of the emission spectra from the sample brass in the split beam mode and the single beam mode at 43 mJ. The delay time is 900 ns and the gate width is 300 ns. (b) Spectroscopic parameters of Cu(i) lines used to determine the plasma temperature.^31^

Here, the peak intensities corresponding to the lines at 465.11 nm, 510.55 nm, 515.32 nm, and 521.82 nm of Cu(i) are utilized for the evaluation of *T*_p_. As shown in Fig. 4(a), for the sample brass, an obvious enhancement of spectral intensity is also found at the delay time of 900 ns in the spectral range of 461–528 nm. The relevant spectroscopic constants for the copper transitions are listed in Fig. 4(b), taken from the NIST database.^31^

**Fig. 5:**
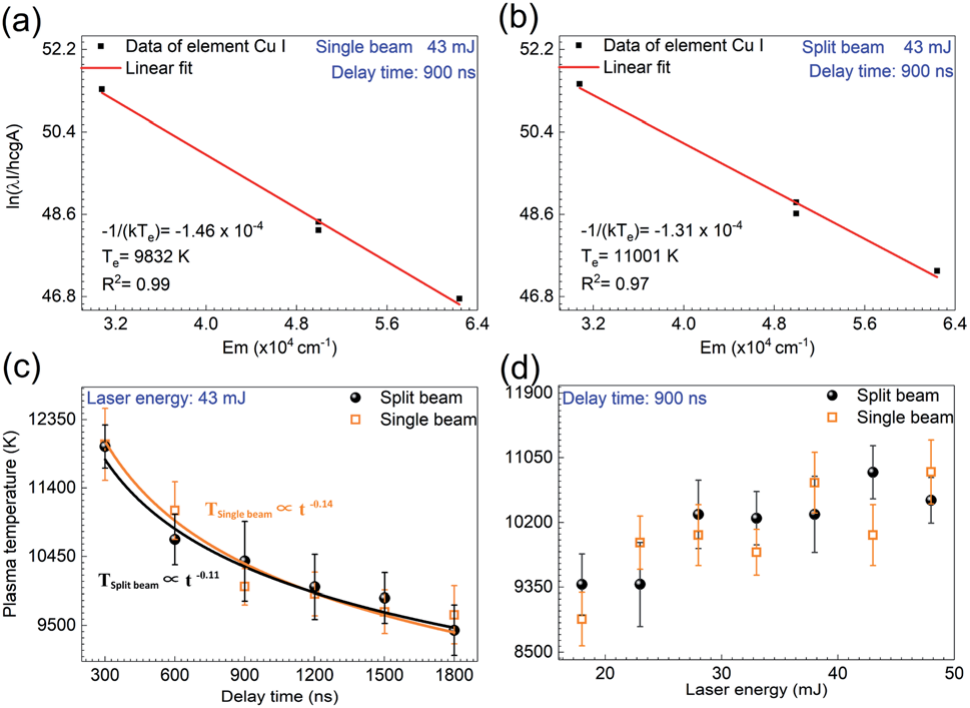
Boltzmann plots for plasma temperature determination by using Cu atomic lines at 465.2 nm, 510.55 nm, 515.32 nm, and 521.82 nm in (a) the single beam mode and (b) the split beam mode at 43 mJ. (c) Comparison of the time-resolved plasma temperature in the single beam mode and the split beam mode at 43 mJ. (d) Comparison of the variation of plasma temperature with the laser energy in the single beam mode and the split beam mode at the delay time of 900 ns. The error bar indicates the data deviation among 10 measurements under the same experimental condition.

Fig. 5(a) and (b) show the Boltzmann plots for the plasma temperature in the single beam mode and split beam mode, respectively, where the laser energy is 43 mJ and the delay time is 900 ns. The plasma temperature is calculated to be 9832 K for the single beam mode and 11 001 K for the split beam mode. With the laser energy unvaried, the plasma temperature is examined at other delay times. Fig. 5(c) displays the temporal evolution of plasma temperature. The value of temperature in both shooting modes is obtained by averaging over 6 repeated calculations, where each calculation is based on five measurements of time-resolved spectra. And the standard deviation of these 6 calculations is considered as the error bar. In order to acquire more reliable plasma temperature, the range of delay time was set as 0–1800 ns, where the selected Cu(i) lines with higher signal-to-noise ratio were obtained. As shown here, the plasma temperatures under the two shooting modes have little difference and they both decline from about 12 000 K to 9500 K within the 1500 ns duration of the laser plasma. The temporal evolution of plasma temperature indicates that the plasma cooling exhibits a strong dependence on delay time (*t*). Different from the adiabatic expansion approximation *T*_p_ ∝ *t*^−3^, the plasma temperature decay is proportional to *t*^−0.11^ for the single beam mode and *t*^−0.14^ for the split beam mode.^32^ This decay of plasma temperature is due to the rapid conversion from thermal energy into kinetic energy, with the plasma attaining a high expansion velocity.^31^ A similar behavior with deviation from *T*_p_ ∝ *t*^−3^ has been noted by other groups.^32–35^

When setting the delay time at 900 ns, the plasma temperature was also calculated at other laser energies, with the results depicted in Fig. 5(d). It is seen that under all the laser energies of interest, the plasma temperature is estimated to be 10 121 ± 1619 K within the measurement uncertainty of 16%, which mainly comes from the uncertainties in the transition probabilities and the measurement of the spectral intensities of characteristic lines in the Boltzmann plots.^36,37^ Comparative analysis of the plasma temperatures indicates that both the laser energy and the shooting mode produce little effect on the plasma temperature.

A model for the condition of the laser supported radiation wave has been described, from which the plasma temperature *T*_p_ can be derived according to the following equation:^38^3
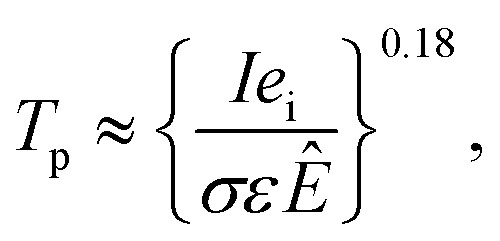
where *I* is the laser irradiance, *e*_i_ is the energy of the laser plasma at the ignition point, *σ* is the Stefan–Boltzmann constant, and *ε* is the emissivity of the plasma. Here, *Ê* represents the correlation coefficient between the internal energy of the fully established plasma and the plasma temperature. The ratio *e*_i_/*Ê* depends on the ionization potentials instead of the atom density. *ε* depends on the initial atom density and degree of ionization at *T*_p_. Since the exponent of 0.18 in eqn (3) is very small, the plasma temperature has little dependence on the laser irradiance, atom density, and ionization potentials of component atoms and ions, which explains the little change in the plasma temperature under various laser energies and the shooting modes.

The electron density is another key parameter that affects LIBS spectral features, which can be determined by the measurement of the Stark broadened line profile. Two important broadening mechanisms are likely to contribute to the line width observed in laser-induced plasma: Doppler broadening and Stark broadening. The contribution from Doppler broadening with Gaussian profile is mainly due to thermal motion of emitting species, which is estimated to be in an order of 0.01 nm.^22^ The Stark broadening with Lorentzian profile dominates the spectral line broadening since the collisions with charged species.^36^ In this work, the emission spectral line is fitted with Voigt function. Through the deconvolution procedure, the FWHM of Lorentzian profile Δ*λ*^Total^_L_ is obtained from the measured line width Δ*λ*^Total^. Then, the Stark-broadened line can be extracted from Δ*λ*^Total^_L_ by simply subtracting the instrumental broadening Δ*λ*^Instrument^_L_ by the relation:

4 Δ*λ*^Stark^_L_ = Δ*λ*^Total^_L_ − Δ*λ*^Instrument^_L_

The contribution from instrumental broadening Δ*λ*^Instrument^_L_ is measured to be about 0.048 nm from emission line of a standard low pressure mercury source.^22^ Then the FWHM of the Stark broadened profile (Δ*λ*^Stark^_L_) scales with the electron density (*N*_e_) as:^37^5
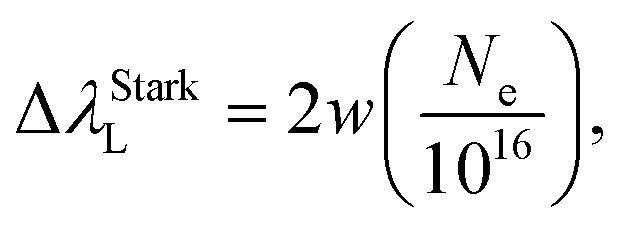
where *w* is the electron width parameter. The value of *w* corresponding to the different plasma temperature is taken from the reference data.^39^ In this study, Cu(i) line at 324.754 nm was chosen for electron density determination. The uncertainties are within 20% for determination of the electron density, which comes from the width measurement, the width deconvolution, and the related parameter *w*.^18^

**Fig. 6:**
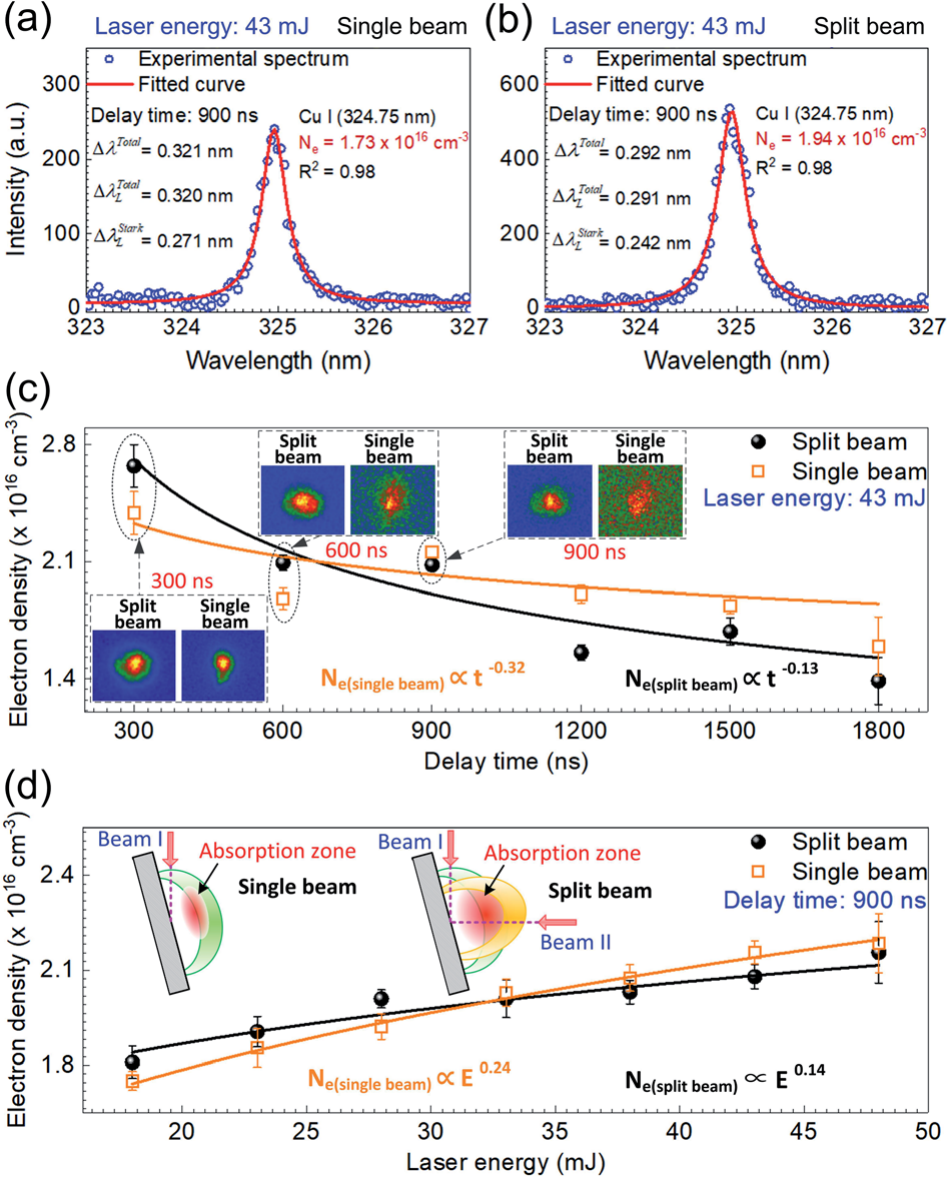
Electron density determination by fitting Voigt function (red solid line) to the experimental spectra (blue circle) of Cu(i) line (324.754 nm) under (a) the single beam mode and (b) the split beam mode at 43 mJ. (c) Comparison of the time-resolved electron density in the single beam mode and the split beam mode at 43 mJ. (d) Comparison of the variation of electron density with the laser energy in the single beam mode and the split beam mode at the delay time of 900 ns. The gate width is set at 300 ns in (a)–(d). The error bar in (c) and (d) indicates the data deviation within 10 measurements under the same experimental condition. Fast images of laser-induced Cu plasma with the gate width of 300 ns at the delay time of 300 ns, 600 ns, and 900 ns in both shooting modes are inset in (c) and the schematic diagrams of laser ablation model in both shooting modes are inset in (d).

The experimental result (blue circle) at 324.754 nm shown in Fig. 6(a) fits fairly well with the typical Voigt profile (red solid line) under the single beam mode at the laser energy of 43 mJ. Here the delay time was also set at 900 ns. In this case, the electron density is estimated to be 2.10 × 10^16^ cm^−3^, where Δ*λ*^Total^, Δ*λ*^Total^_L_, Δ*λ*^Stark^_L_ equal 0.343 nm, 0.342 nm, and 0.293 nm, respectively. The electron densities on the order of 10^16^ cm^−3^ are observed in the single and split laser beam modes, agreeing well with those reported in laser-produced copper plasma.^36^

The temporal evolution of the electron density was examined in both shooting modes when the laser energy was fixed at 43 mJ, with the results shown in Fig. 6(c). Here, the symbols in both shooting modes represent the averaged value of 6 repeated calculations and each calculation is based on five measurements of time-resolved spectra. The standard deviation of these 6 repeated calculations is considered as the error bar. Similar to the trend of plasma temperature, the variation of electron density with the delay time is observed to decay with a relationship *N*_e_ ∝ *t*^−0.25^ for the single beam mode and *N*_e_ ∝ *t*^−0.33^ for the split beam mode. In the case of single beam, the density decreases from 2.39 × 10^16^ cm^−3^ to 1.50 × 10^16^ cm^−3^, whereas, in the case of split beam, it varies from 2.67 × 10^16^ cm^−3^ to 1.39 × 10^16^ cm^−3^, as the delay time increases from 0 to 1800 ns. Harilal *et al.* have also reported such a behavior using 1.06 μm radiation pulses for the sample graphite, in which the electron density varied from 3.6 × 10^17^ cm^−3^ to 1.5 × 10^17^ cm^−3^ during the plasma expansion.^33^ It is also worth noting that the decrease in electron density for the split beam mode is faster than that for the single beam mode. In the early phase of plasma decay, the laser plasma has a higher electron density in the split beam mode than in the single beam mode because of the higher laser irradiance. However, in the late phase of plasma decay, the electron density in the split beam mode is overwhelmed by that in the single beam mode. As insets in Fig. 6(c), the fast images of laser-induced Cu plasmas with the gate width of 300 ns at the delay time of 300, 600, and 900 ns in both shooting modes, show a larger volume and higher brightness in the spit beam mode, which suggests that the plasma plume undergoes a faster expansion process under the condition of split beam. This behavior accelerates the reduction of the electron density, which is mainly responsible for the lower electron density in the late phase of plasma decay in the split beam mode. Then, this relatively low electron density partially results in the decreasing enhancement effect of spectral intensity in the late phase of plasma decay, as shown in Fig. 3.

With the delay time set at 900 ns, the dependence of electron density on the laser energy was examined in the range of 18–48 mJ. In both the shooting modes, an identical rising tendency of electron density with the laser energy is presented in Fig. 6(d). The electron density in the single beam mode and the split beam mode is proportional to *E*^0.14^ and *E*^0.24^, respectively. In detail, the electron density varies as 1.75 × 10^16^ cm^−3^–2.18 × 10^16^ cm^−3^ in the single beam case and 1.81 × 10^16^ cm^−3^–2.16 × 10^16^ cm^−3^ in the split beam case. The increase in the electron density with the laser energy is due to the stronger interaction between sample and laser pulse, where more excited species, ions and free electrons are generated because more mass is ablated with increasing laser energy, under the condition that the laser energy is far below the threshold of saturation in the mass removal due to the plasma shielding effect.^37^

Additionally, from Fig. 6(d), it follows that the electron density in the split beam mode is higher than that in the single beam mode when the laser energy is no more than 33 mJ. But with the laser energy exceeding 33 mJ, the electron density in the split beam mode becomes smaller, compared to the case in the single beam mode. The variation of electron density with the laser energy under different shooting modes can be explained by the plasma shielding effect. When the incident irradiance is not much higher than the breakdown threshold in the initial phase of laser pulse, the LIBS plasma is typically in the laser-supported detonation (LSD) wave regime. Here, the shock front is strong enough to heat the gas and almost all the laser energy is absorbed as the input of laser energy is increased. The main physical processes involved are heat conduction, melting, and vaporization of the sample.^1^ At a lower laser energy, the vapors generated are not so hot or dense, which results in a weak absorption effect under both shooting modes. Thus, either the single beam or the split beams could pass nearly unattenuated through vapors and touch the sample surface. Since the excitation process in the spit beam mode is more efficient than that in the single beam mode due to the higher laser irradiance under the same laser energy, a higher electron density is observed under the split beam scheme at the laser energy below 33 mJ.

However, as the laser energy rises, the plasma density increases to a high value, and the plasma behaves like an optically thick medium and shields the target surface. Then an effective absorption zone is formed, as shown in the schematic diagrams of laser ablation model inset in Fig. 6(d). It has been reported that the thickness of absorption zone is inversely proportional to the angle of incident laser beam.^19^ In the single beam mode, a large incident angle of around 85° gives rise to a thin absorption zone. It follows from the inset on the left that the incident path of beam I indicated by the pink dash line passes through a less area of the absorption zone. Thus, the weak absorption effect ensures most of the single beam reaches the sample surface. In the split beam mode, one beam is split into two branches. The right inset shows that the horizontal beam II passes through the main area of the thick absorption zone. Therefore, a considerable part of the horizontal beam II is blocked by the plasma and the increment of sample vaporized by beam II declines as the laser energy increases. The increase in electron density with the laser energy in the split beam mode is decelerated due to the increasing absorption effect, which is responsible for the lower electron density in the split beam mode, when the laser energy is above 33 mJ. Due to the plasma shielding, the increasing absorption effect with the laser energy in the split beam mode also accounts for the optimal enhancement performance observed at the laser energy of 28 mJ in Fig. 3.

As mentioned above, the use of the emission spectra for the measurement of plasma temperature is based on the assumption of LTE. Determination of electron density using stark broadening does not require LTE, which assumes that the population and depopulation of atomic and ionic states occur predominantly by collision rather than by radiation. This collision process requires an electron density which is sufficient to ensure the high collision rate. And the corresponding lower limit of the electron density is given by the McWhirter criterion:^4^

6 *N*^Lower^_e_ (cm^−3^) ≥ 1.6 × 10^12^*T*_p_^1/2^(Δ*E*)^3^,

where Δ*E* (eV) is the energy difference between the two states. In our case, the maximum Δ*E* is 3.82 eV for Cu(i) at 324.75 nm, *T*_p_ is about 10 000 K, and *N*_e_ is around 2.26 × 10^16^ cm^−3^. The low limit of electron density was calculated to be 8.85 × 10^15^ cm^−3^ from eqn (6). Under the condition of 2.26 × 10^16^ cm^−3^ > 8.85 × 10^15^ cm^−3^, the electron density determined in our experiment satisfies the LTE condition.

## 4. Conclusions

In summary, a single-beam-splitting approach was employed to enhance the spectral intensity of LIBS under the extreme condition of laser beam grazing the surface of non-flat samples. The time-resolved characteristics of laser induced plasma were comparatively investigated under the single beam mode and split beam mode.

From time-resolved spectra of aluminium alloy and brass samples, it is observed that the single-beam-splitting approach allows for stronger signal intensity and longer plasma emission lifetime because of the higher laser irradiance. Examining the signal enhancement factors of Al(i) and Cu(i) lines under different laser energies shows that a similar tendency of enhancement factor with the delay time is observed for all the cases. This enhancement factor first rises because the contribution of the increased laser irradiance in the split beam mode to the atomic spectra gradually grows in the early phase of plasma decay, and then the factor falls in the late phase of plasma decay due to the plasma cooling and the relatively low electron density in the faster plasma expansion in the split beam mode. For Al plasma, the remarkable feature is that the delay time at which the maximum of enhancement factor occurs is later than that of Cu plasma under the same laser energy, which is largely attributed to its stronger spectral intensity and longer lifetime as result of the different atomic structures and different energy levels of the observed emission lines.

It is also found that the temporal evolution of plasma temperature almost remains unchanged owing to the weak dependence of plasma temperature on the laser irradiance, when changing the shooting mode. However, examining the electron density shows that the electron density decreases faster in the split beam mode as the plasma decays, which mainly results from the faster expansion process of plasma. Moreover, the electron density increases slower with the laser energy in the split beam mode, which is attributed to the plasma shielding effect.

Our results show that single beam splitting is an effective way to enhance the signal intensity of LIBS even under the laser energy below 60 mJ. The time-resolved characteristics of LIBS on the non-flat samples in the single beam mode and split beam mode provide us a valuable reference for highly efficient utilization of the single-beam-splitting approach in LIBS measurement of irregular targets, especially for the samples composed of hard materials with macroscopic uneven surface.

## Conflicts of interest

There are no conflicts to declare.

## Supplementary Material
